# Supplementary material for: Elevated serum magnesium lowers calcification propensity in Memo1-deficient mice
Source: PLoS One. 2020 Jul 24;15(7):e0236361. doi: 10.1371/journal.pone.0236361 (PMC7380890; doi:10.1371/journal.pone.0236361)
Supplement: S1 File — (PDF) [file pone.0236361.s001.pdf]

- Trpv5

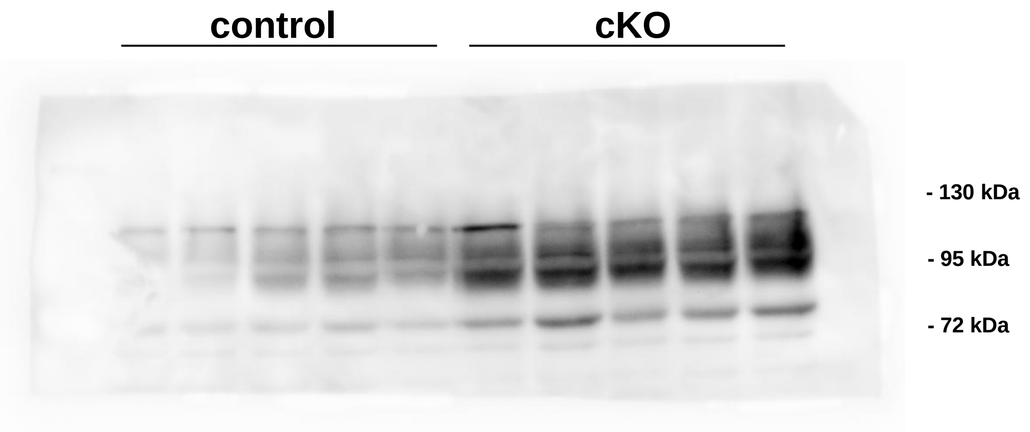

- PMCA

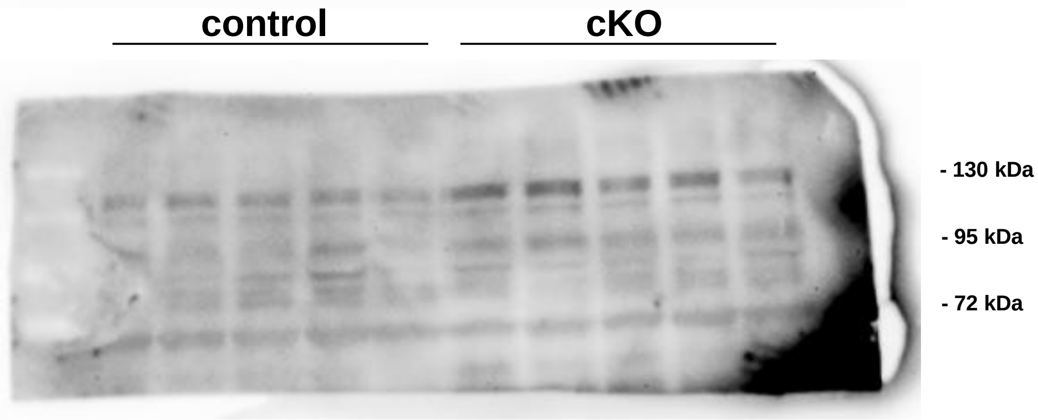

- Ponceau S

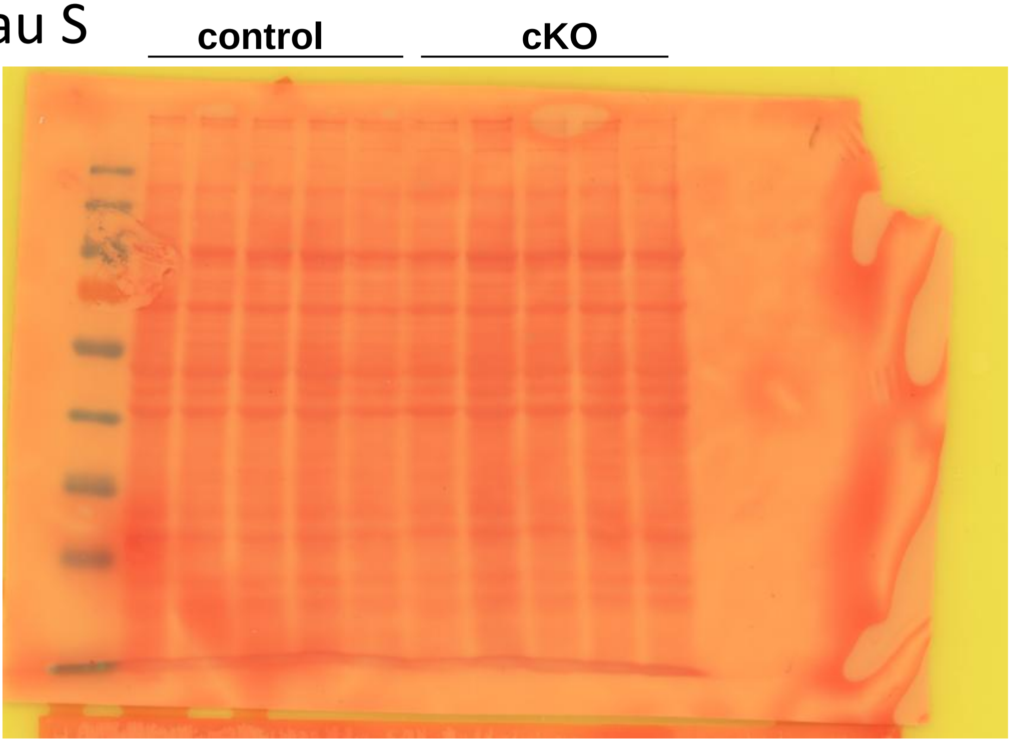

Figure 1C

• Calbindin D28K

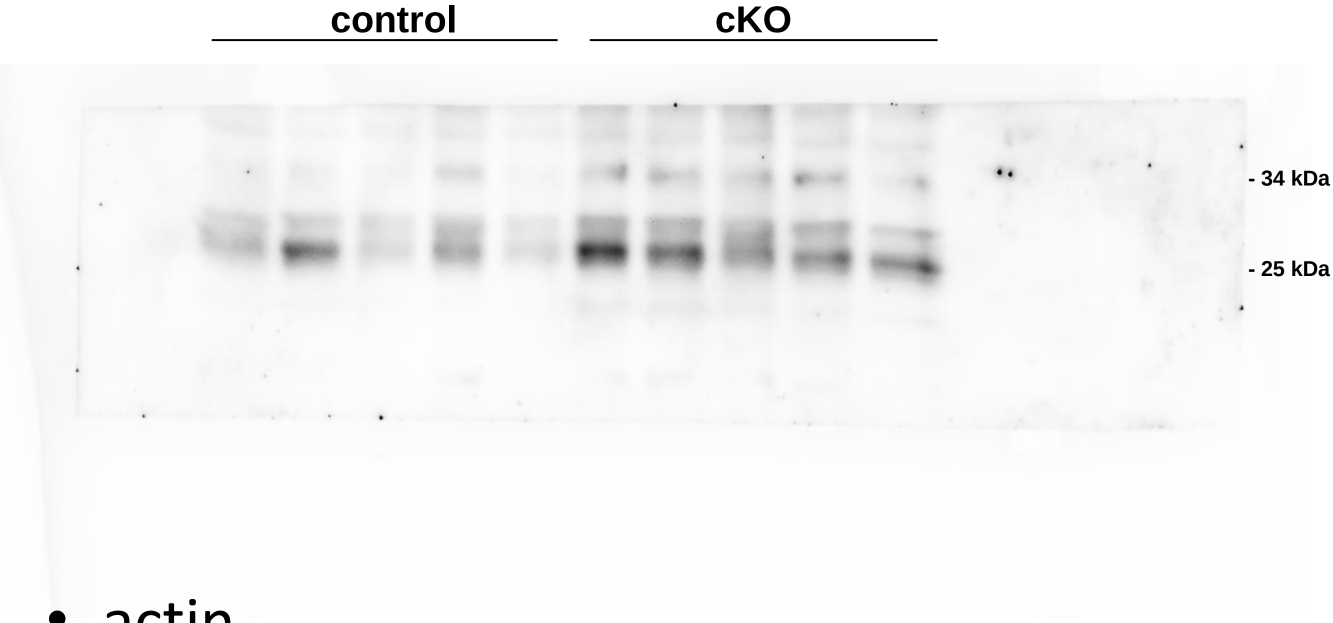

• actin

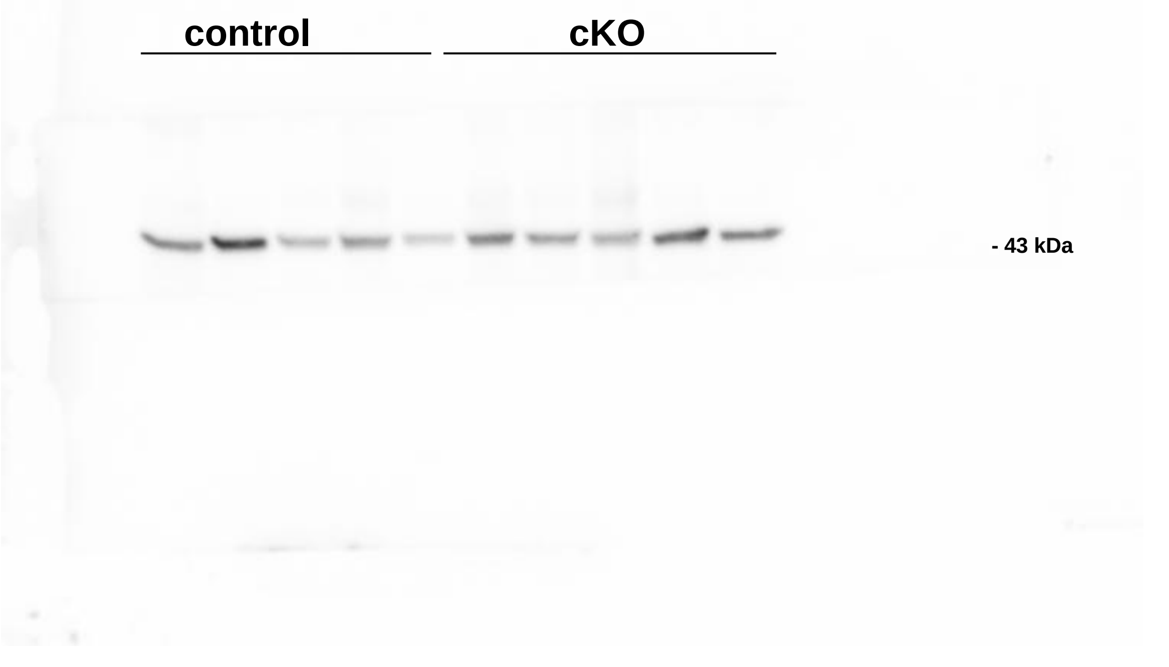

Figure 1D

- NCX1

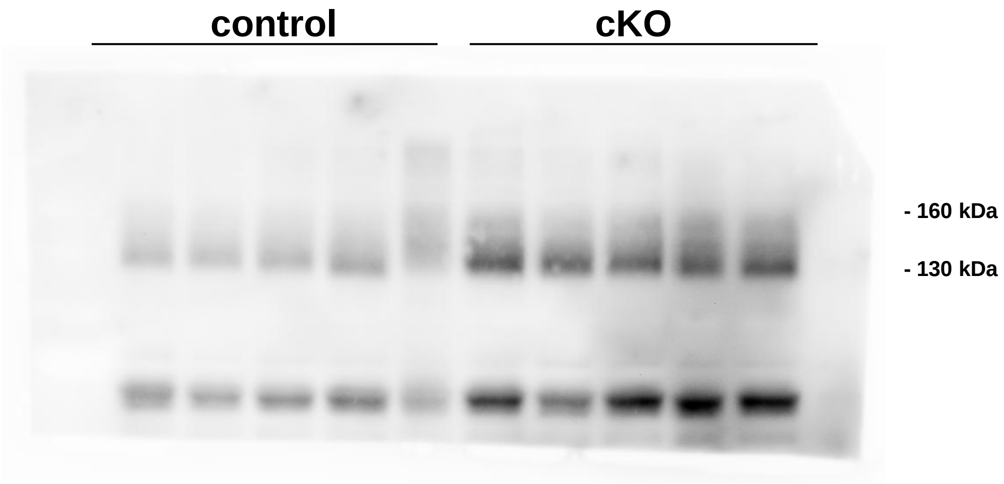

- ponceau S

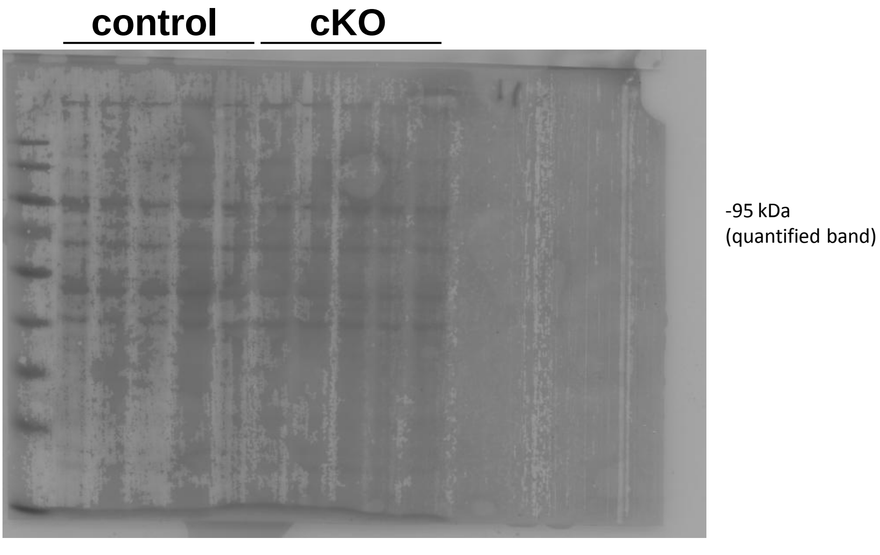

Figure 1E

- NaPi2a

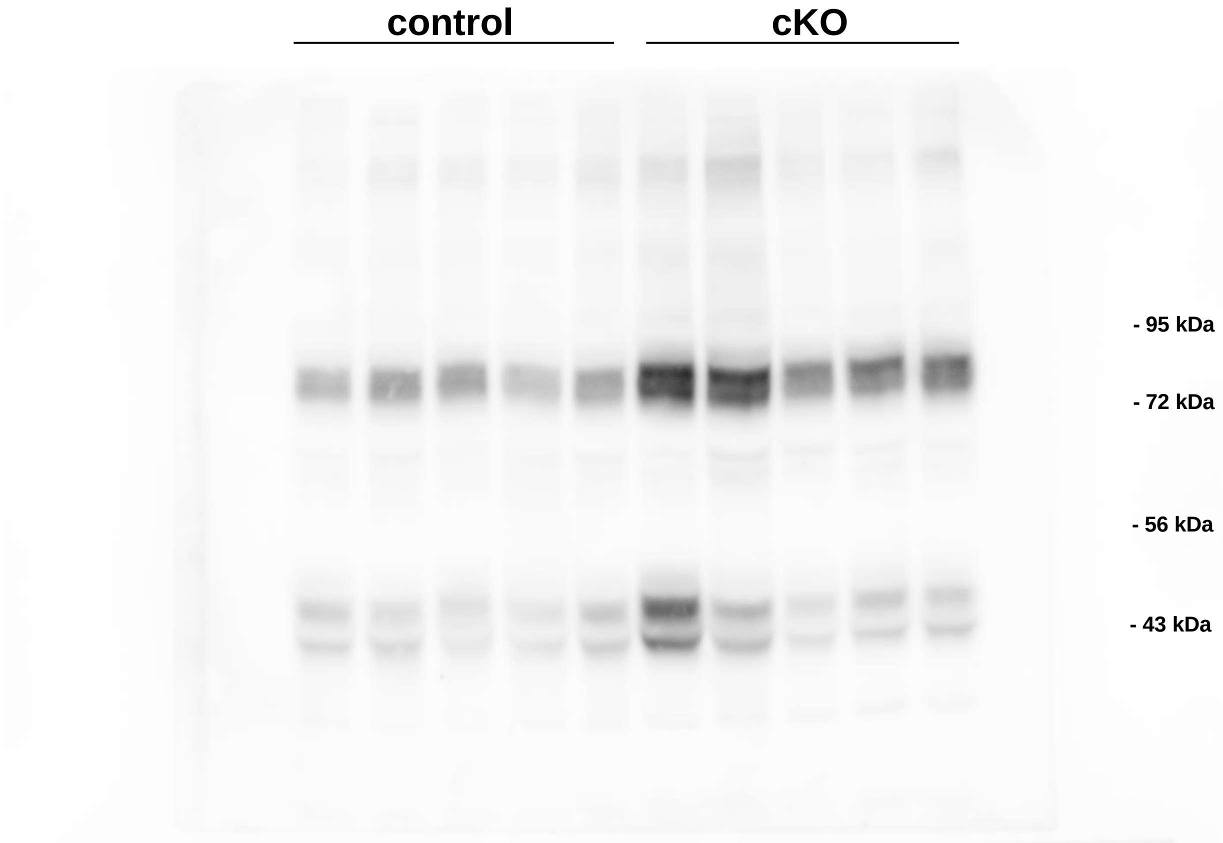

- Ponceau S

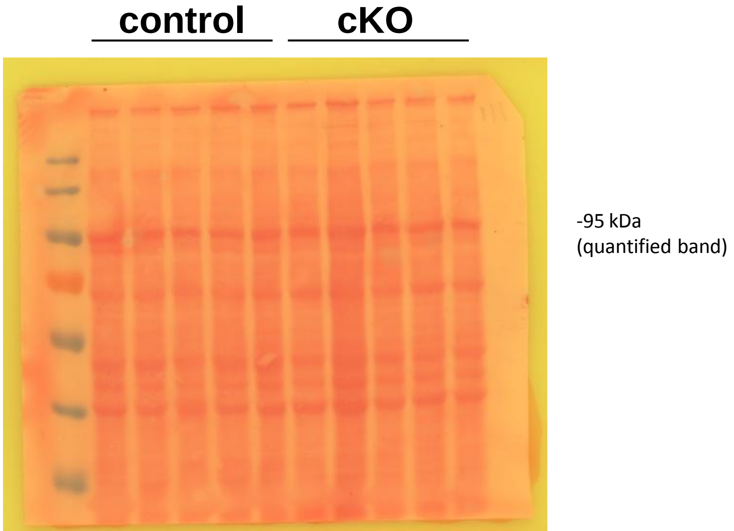

Figure 2B

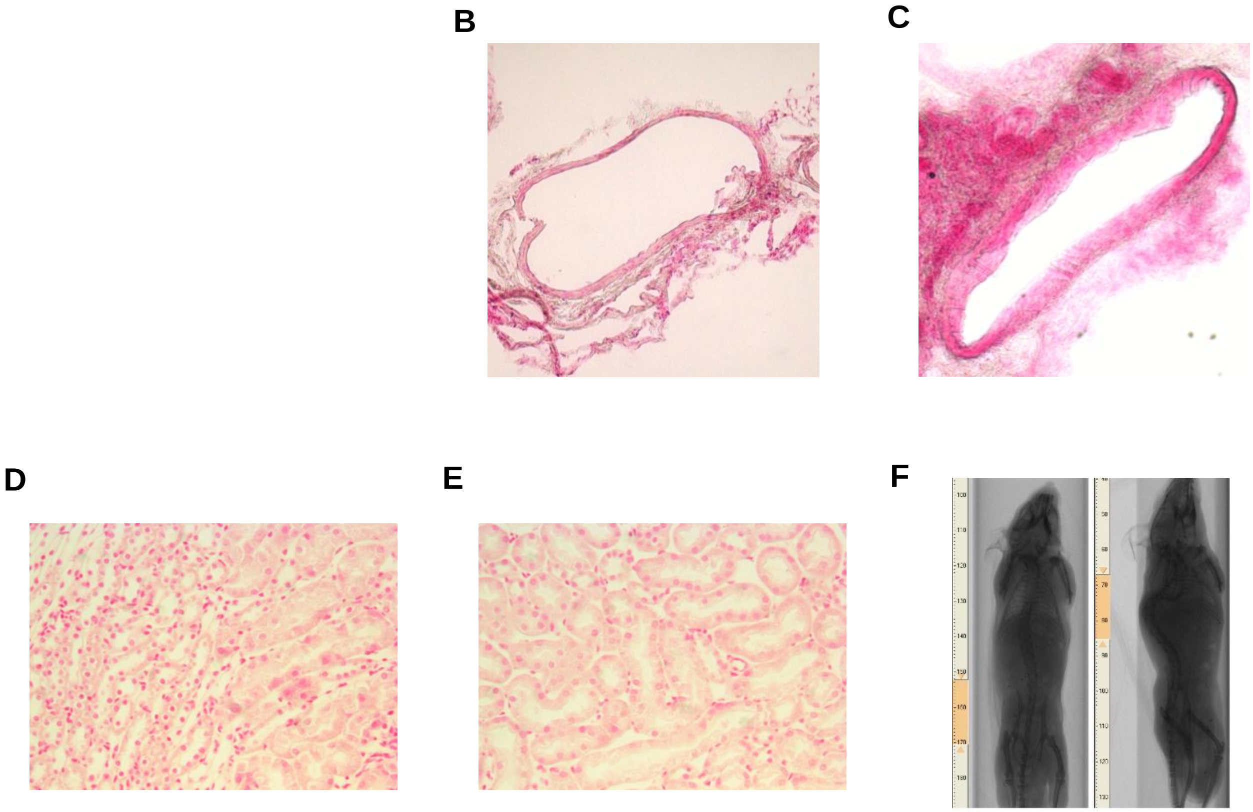

Figure 4

A

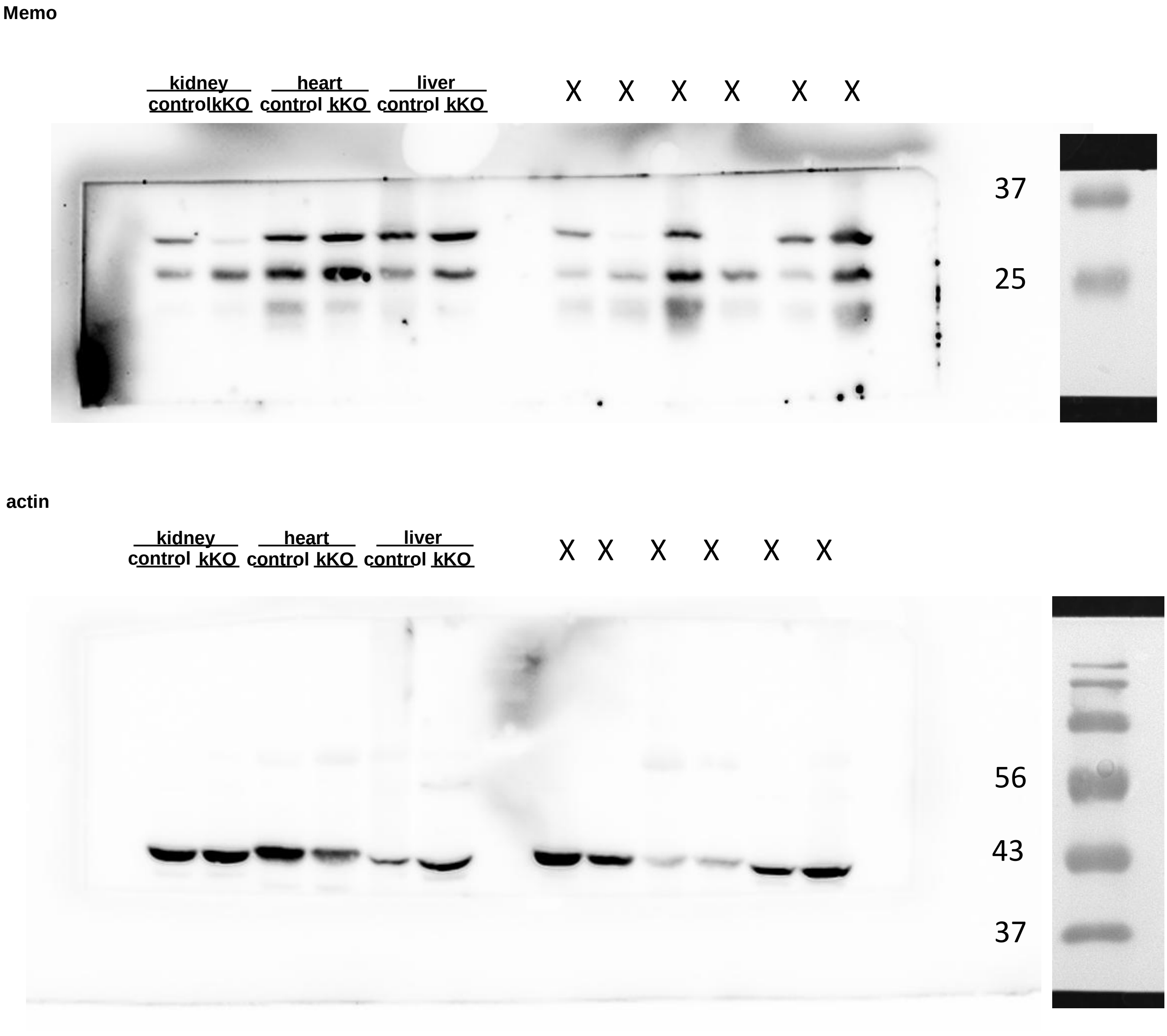
Memo
kidney
control kKO
heart
control kKO
liver
control kKO
X X X X X X
37
25
actin
kidney
control kKO
heart
control kKO
liver
control kKO
X X X X X X
56
43
37

Figure 7A
